# Evaluation of the Diagnostic Accuracy of Clinical Examination for Diagnosing Medial Meniscal Injuries Using Arthroscopy

**DOI:** 10.7759/cureus.89026

**Published:** 2025-07-29

**Authors:** Muhammad Mannan, Sarmad Khalil, Muhammad Awais Iqbal, Faisal Karim, Malik Haroon Hameed, Nayan Shrivastava, Muhammad Tayyab, Rehan Raza Shan

**Affiliations:** 1 Trauma and Orthopaedics, Ghurki Trust Teaching Hospital, Lahore, PAK; 2 Trauma and Orthopaedics, Sheikh Zayed Medical College Rahim Yar Khan, Rahim Yar Khan, PAK; 3 Orthopaedic Surgery, University Hospitals Birmingham NHS Foundation Trust, Birmingham, GBR; 4 Trauma and Orthopaedics, Razia Saeed Hospital, Multan, Multan, PAK; 5 Trauma and Orthopaedics, Queen Elizabeth Hospital Birmingham, Birmingham, GBR; 6 Trauma and Orthopaedics, The Dudley Group NHS Foundation Trust, West Midlands, GBR; 7 Medicine, Khyber Medical University, Peshawar, PAK; 8 Trauma and Orthopaedics, Kettering General Hospital, Kettering, GBR; 9 Orthopaedics, Sancheti Institute for Orthopedics and Rehabilitation College of Physiotherapy, Pune, IND; 10 Trauma and Orthopaedics, University Hospitals Birmingham NHS Foundation Trust, Birmingham, GBR; 11 Trauma and Orthopaedics, Hayatabad Medical Complex Peshawar, Peshawar, PAK; 12 Trauma and Orthopaedics, Bradford Teaching Hospitals NHS Foundation Trust, Bradford, GBR; 13 Trauma and Orthopaedics, Heartlands Hospital, University Hospitals Birmingham NHS Foundation Trust, Birmingham, GBR

**Keywords:** clinical examination, diagnosis, diagnostic accuracy, knee injury, medial meniscus injury, mri

## Abstract

Introduction

Traditional manoeuvres such as the McMurray's test and the Apley compression test have low diagnostic accuracy when performed in isolation. However, by combining these tests, higher accuracy can be found. The superficial anatomy of the knee enables diagnosis of the injury through a thorough history and physical examination.

Objective

The objective of this study is to determine the diagnostic accuracy of a combination of physical examination tests, such as Apley’s test, McMurray’s test, and joint line tenderness, for diagnosing medial meniscal injuries using arthroscopy as the gold standard.

Materials and methods

This cross-sectional study was conducted over a duration of 12 months, from February 15, 2021, to February 15, 2022, in the Department of Orthopedics at Ghurki Trust Teaching Hospital, Lahore, Pakistan. A total of 185 patients who met the inclusion criteria were enrolled in the study after obtaining approval from the hospital ethics committee. Informed consent was obtained from each patient or their attendant prior to participation.

All clinical examinations and arthroscopic procedures were performed by a single experienced consultant to ensure consistency. The clinical examination included Apley’s test, joint line tenderness test, and McMurray’s test, and findings were recorded as positive based on predefined operational definitions. Arthroscopic evaluations were performed under spinal anaesthesia during definitive surgical procedures, and medial meniscal injury was diagnosed in accordance with the operational definition.

Results

The mean age of patients was 48.54 ± 11.49 years, with a minimum and maximum age of 18 and 65 years. There were 119 (64.3%) male and 66 (35.7%) female cases. On arthroscopy, the medial meniscal injury was diagnosed in 127 (68.65%) of the cases, and on clinical examination, it was found in 122 (65.95%) of the cases. The sensitivity, specificity, positive predictive value (PPV), negative predictive value (NPV), and diagnostic accuracy of the clinical examination were 90.55%, 87.93%, 94.26%, 80.95% and 89.73%, respectively.

Conclusion

It is concluded that the diagnostic accuracy of clinical examination for the diagnosis of medial meniscal injury was good when arthroscopy was taken as the gold standard. Hence, in the future, we can rely on clinical examination for diagnosis for patients who have a fear of undergoing arthroscopy. Arthroscopy can be used to treat these injuries in patients willing to undergo treatment. With better diagnostic accuracy, we can treat these cases timely manner to avoid further degenerative changes of the cartilage in the medial compartment of the knee.

## Introduction

Injuries to the knee are among the most prevalent types of injuries. Injuries to the knee joint are on the rise, both as a result of sport and trauma; they are more frequently observed in young people who are physically active [1,2]. An injury to the meniscus disrupts the normal mechanics and stability of the knee joint, leading to an unstable knee that makes it difficult for a person to carry out regular daily activities [3,4]. As a result, these injuries ought to be precisely recognized and addressed to the greatest extent as soon as possible, either through surgical or conservative means [5].

In most patients, the clinical symptoms can include locking, clicking, joint swelling, and claudication [6]. Numerous physical tests have been described, but their diagnostic accuracy is often questioned. The McMurray's test and the Apley compression test are two examples of traditional techniques that, when performed in isolation, have a low diagnostic accuracy. However, by combining these tests, it is possible to achieve a better level of accuracy [7].

Although several studies are available, there is variation in the reported sensitivity and specificity of clinical examination, with sensitivity ranging from 80.77% to 100% [8] and specificity ranging from 57.1% to 78.57% [8,9]. At Ghurki Trust Teaching Hospital, Lahore, Pakistan, a large number of patients present with such conditions. If the clinical examination findings provide a precise diagnosis of medial meniscus injury and are in concordance with arthroscopic findings, then in the future, clinical examination could be relied upon for diagnosis in patients who fear undergoing arthroscopy. Arthroscopy can be used to treat these injuries in patients willing to undergo the procedure. With better diagnostic accuracy, these cases can be treated in a timely manner to avoid further degenerative changes in the cartilage of the medial compartment of the knee.

## Materials and methods

Following the acceptance of the synopsis, this cross-sectional study was conducted in the Department of Orthopedics at Ghurki Hospital in Lahore for 12 months, commencing on February 15, 2021, and concluding on February 15, 2022. This study was approved by the Research Evaluation Unit of the College of Physicians and Surgeons Pakistan (CPSP) under approval number CPSP/REU/OSG-2019-080-2113. Overall, 185 patients took part in the research study. To determine the sample size, a 51.43% prevalence of medial meniscus damage was used. Furthermore, the clinical examination's sensitivity and specificity were calculated to be 80.77% and 78.57%, respectively. The margin of error for sensitivity and specificity in the study was kept at 8% and 8.5%, respectively, based on a 95% confidence level. A predicted sample size of 185 was calculated using a single population proportion formula. Specifically, a non-probability consecutive sampling technique was employed, whereby all eligible patients who met the inclusion criteria and presented during the defined study period were recruited.

The study comprised patients who reported symptoms consistent with medial meniscal injury, as defined by the operational definition. Patients of either gender, aged 18 to 65 years, came with symptoms. Patients who had previously undergone knee surgery, were unable to walk due to a major illness, had degenerative changes, loose bodies, or fractures apparent on plain radiographs, or had previously received non-operative treatment for meniscal injuries were excluded from the study.

Patients who met the enrolment criteria and gave informed consent were included in the study. The researcher was in charge of acquiring demographic information such as age, gender, and relevant clinical data for the study. Clinical examinations and arthroscopies were carried out by one qualified consultant. The consultant was not blinded to the patient’s history or imaging findings, which may represent a potential source of observer bias. The clinical examination results, which included the Apley's test, joint line tenderness test, and McMurray's test, were evaluated using the previously established operational criteria. Apley’s test was considered positive if compression of the knee with rotation elicited joint line pain. McMurray’s test was interpreted as positive when a palpable or audible click was felt during knee rotation along with pain on the medial joint line. Joint line tenderness was labelled positive if localized tenderness was elicited upon palpation of the medial joint line. During critical procedures, a diagnostic arthroscopy, which is considered the gold standard, was conducted while under spinal anaesthesia. If tears were spotted and probed in accordance with operating norms, a medial meniscus damage was diagnosed. All procedures were performed by a consultant with documented expertise in knee arthroscopy. However, interobserver variability was not assessed, as all assessments were performed by the same individual.

The researcher employed a methodical proforma to record all acquired data. The data input and analysis operations were carried out using IBM SPSS Statistics for Windows, Version 24 (Released 2017; IBM Corp., Armonk, New York, United States). The mean and standard deviation were calculated for quantitative variables such as age and body mass index, whereas frequencies and percentages were provided for categorical variables such as gender and medial meniscal injury diagnosis (based on clinical examination and arthroscopy). To determine the diagnostic accuracy, a 2 by 2 contingency table was constructed. The calculated metrics included sensitivity, specificity, positive predictive value (PPV), negative predictive value (NPV), false positive rate, and false negative rate. To adjust for potential effect modifiers, the data were stratified according to age, gender, and obesity. Following that, a post-stratification analysis was performed using a p-value of 0.05 or less to determine statistical significance. Effect size was not separately calculated, but the diagnostic accuracy measures served as the primary indicators of effect.

## Results

The mean age of patients was 48.54 ± 11.49 years, with a minimum and maximum age of 18 and 65 years. There were 57 (30.81%) patients who were 18-40 years old, and 128 (69.19%) patients were 41-65 years old. There were 119 (64.3%) male and 66 (35.7%) female patients. The mean BMI was 28.35 ± 3.47 with a minimum and maximum BMI of 21 and 36.80. There were 61 (32.97%) obese and 124 (67.03%) non-obese patients (Table 1).

**Table 1:** Demographic and Baseline Characteristics of the Patients (n = 185)

Variable	Category	Frequency (%)/Mean ± SD
Age (years)	18-40	57 (30.81)
	41-65	128 (69.19)
Mean age	-	48.54 ± 11.49
Gender	Male	119 (64.3)
	Female	66 (35.7)
BMI	Obese	61 (32.97)
	Non-obese	124 (67.03)
Mean BMI	-	28.35 ± 3.47

On arthroscopy, the medial meniscal injury was diagnosed in 127 (68.65%) of the cases, and on clinical examination, it was found in 122 (65.95%) of the cases. There were 115 cases who had medial meniscal injury on both arthroscopy and clinical examination; 51 cases did not have medial meniscal injury. There were seven false positives and 12 false negatives. The sensitivity, specificity, PPV, NPV, and diagnostic accuracy of the clinical examination were 90.55%, 87.93%, 94.26%, 80.95% and 89.73%, respectively (Table 2).

**Table 2:** Diagnostic Results of Clinical Examination Versus Arthroscopy

Diagnosis Status	Arthroscopy: Yes	Arthroscopy: No	Total
Clinical exam: Positive	115	7	122
Clinical exam: Negative	12	51	63
Total	127	58	185

When stratified by age, clinical examination showed slightly higher diagnostic accuracy among the younger age group (18-40 years), with an accuracy of 92.98%, compared to 88.28% in the older group (41-65 years). Sensitivity and NPV were also notably higher in the younger group.

Gender-based analysis showed comparable results, with slightly better performance among females (accuracy 90.91%) than males (accuracy 89.08%). Both groups had high sensitivity and PPV, indicating that clinical examination was effective regardless of gender.

When stratified by BMI, obese patients had 100% sensitivity and NPV, but lower specificity (80.00%) and PPV (83.78%). In contrast, non-obese individuals had lower sensitivity (87.50%) but notably higher specificity (96.43%) and PPV (98.82%). These findings suggest that clinical examination may yield more false positives in obese individuals, but is otherwise highly accurate across both groups (Table 3).

**Table 3:** Diagnostic Accuracy Stratified by Age, Gender, and Body Mass Index (BMI) PPV: positive predictive value; NPV: negative predictive value

Variable	Category	Sensitivity	Specificity	PPV	NPV	Accuracy
Age	18-40 yrs	94.59%	90.00%	94.59%	90.00%	92.98%
	41-65 yrs	88.89%	86.84%	94.12%	76.74%	88.28%
Gender	Male	90.00%	87.18%	93.51%	80.95%	89.08%
	Female	91.49%	89.47%	95.56%	80.95%	90.91%
BMI	Obese	100.00%	80.00%	83.78%	100.00%	90.16%
	Non-obese	87.50%	96.43%	98.82%	69.23%	89.52%

Overall, the findings support that clinical examination, when all components (Apley’s test, joint line tenderness, and McMurray’s test) are considered together, offers high diagnostic accuracy and can serve as a dependable alternative to arthroscopy, especially in settings where access to surgical intervention is limited or in patients hesitant to undergo invasive procedures.

## Discussion

A multitude of clinical trials and diagnostic investigations have been undertaken to enhance doctors' capacity to accurately detect knee issues. Ruptured meniscus or ligamentous structures in the knee result in considerable discomfort and incapacity, necessitating immediate and precise treatment and care [10]. Given the severe ramifications of meniscus injuries in patients, particularly those sustained during physical activity, prompt and precise identification of these injuries is crucial. The need for surgery after a meniscus injury is mostly dictated by the initial physical examination and supplementary diagnostic assessments.

Joint line discomfort and effusion are indicative of meniscal damage. Palpation of the knee along the axis may provide inconclusive findings in forecasting meniscus tears associated with acute anterior cruciate ligament (ACL) injuries, with a specificity of 34.5% and sensitivity of 44.9% for internal axis palpation, and a specificity of 49.1% and sensitivity of 57.6% for external axis palpation [11].

When the ACL is intact, discomfort along the joint line more reliably (77%) indicates meniscal tears. The primary assessments necessary for diagnosing individuals with such symptoms include joint boundary palpation, the McMurray's test, the Apley grind test, and the Thessaly's test. Arthroscopy is the definitive diagnostic method for severe damage to the knee joint. Arthroscopy, while highly precise, is an invasive and costly procedure necessitating hospitalization and general or regional anesthesia [12].

A noninvasive diagnostic tool that does not require an intra-articular approach might mitigate the dangers associated with arthroscopy and decrease the annual volume of the 1,200 knee arthroscopies performed. While the diagnostic value of a physical examination conducted by a seasoned physician has been assessed in several studies, further research is necessary to determine its superiority over other diagnostic approaches [13].

In the current study, which was conducted over a period of 12 months, the mean age was higher than in a previous study, which reported a mean age of 29.13 ± 7.37 years (range: 16-54), though both studies had a similar gender ratio, i.e., 108 males and 12 females [14]. In the current study, the sensitivity, specificity, PPV, NPV, and diagnostic accuracy of clinical examination were 90.55%, 87.93%, 94.26%, 80.95%, and 89.73%, respectively. Our findings are consistent with other studies that reported higher diagnostic accuracy, with a sensitivity of 80.77%, a specificity of 78.57%, a PPV of 87.5%, and an NPV of 68.75% [8]. A local study reported clinical examination for diagnosis of medial meniscus injury to have a sensitivity of 100%, specificity of 57.1%, and overall diagnostic accuracy of 87.23% [9].

A recent study assessed the accuracy of clinical examination versus MRI, using arthroscopic evaluation as the gold standard for diagnosing meniscal injuries. Arthroscopic findings revealed 32 meniscal tears, including 28 (87%) in the medial meniscus and four (13%) in the lateral meniscus. The injuries included 10 bucket-handle tears, 17 longitudinal tears, and five of various types (transverse, oblique, radial). The authors identified two false-positive and two false-negative instances when comparing MRI findings to arthroscopy. Sixty-two McMurray's test results were valid, with 15 false positives and nine false negatives. Sixty Apley's test results were accurate, with 16 false positives and 10 false negatives. Seventy-eight Thessaly's test results were valid, with five false positives and three false negatives. The comparison of Thessaly's test findings with those of McMurray and Apley's tests demonstrated statistical significance (p < 0.05). However, comparison of Thessaly's test results with MRI showed no statistical significance (p = 0.151), while comparisons of McMurray and Apley's test results with MRI demonstrated statistical significance (p < 0.01). Consequently, clinical examination conducted by a proficient examiner may possess comparable or even superior diagnostic accuracy relative to MRI for assessing meniscal abnormalities. This research confirms the Thessaly test as a credible clinical assessment for diagnosing meniscal tears.

In 2011, a series of five techniques for meniscal injuries (McMurray, Apley, Childress, and Steinmann 1 and 2) was assessed, and their sensitivity, specificity, accuracy, and likelihood ratios were determined [15]. Each test was performed separately. A total of 152 patients of both genders scheduled for knee videoarthroscopy were assessed blindly by one of five residents at the institution, without knowledge of the clinical data or the surgical indication. The assessments were performed just prior to videoarthroscopy, and findings were recorded in an electronic spreadsheet. The investigation revealed that the five meniscal tests had a sensitivity of 89%, specificity of 42%, accuracy of 75%, positive likelihood ratio of 1.53, and negative likelihood ratio of 0.26. The tests showed individual accuracy ranging from 48% to 53% [15].

The high diagnostic accuracy demonstrated in this study supports the use of a structured clinical examination protocol as a diagnostic tool for medial meniscal injuries. In limited settings or when patients are hesitant to undergo invasive procedures such as arthroscopy, a positive combination of Apley’s test, McMurray’s test, and joint line tenderness can guide clinicians in initiating timely treatment or referring for further imaging or surgical intervention. However, clinical examination should be viewed as a useful tool rather than a definitive diagnostic modality. Confirmation through imaging, such as MRI, or surgical assessment via arthroscopy, remains essential, particularly in ambiguous cases or when surgical intervention is being considered.

Limitations

This study has several limitations that should be considered while interpreting the results. First, it was conducted at a single center, which may limit the generalizability of findings to other settings with different patient populations or examiner expertise. Second, all clinical examinations and arthroscopies were performed by a single consultant, which, while ensuring consistency, may introduce examiner bias and does not reflect real-world variability in clinical skill. The reliance on the examiner’s clinical skill underscores that diagnostic accuracy, to some extent, is personnel-dependent and may not be uniformly replicable without standardized training. Third, the study did not account for interobserver variability or compare results across multiple examiners. Additionally, the operational definition required all three clinical tests to be positive, which may not reflect common clinical practice, where even a single test may guide further management. Finally, this study did not compare clinical examination with other diagnostic modalities such as MRI, which could have provided a more comprehensive assessment of diagnostic utility.

## Conclusions

The diagnostic accuracy of clinical examination for the diagnosis of medial meniscal injury was good when arthroscopy was taken as the gold standard. In the future, we can rely on clinical examination for diagnosis for patients who have a fear of undergoing arthroscopy. Arthroscopy can be used to treat these injuries in patients willing to undergo treatment. With better diagnostic accuracy, we can treat these cases timely manner to avoid further degenerative changes of the cartilage in the medial compartment of the knee.

The high diagnostic accuracy demonstrated in this study supports the use of a structured clinical examination protocol as a main diagnostic tool for medial meniscal injuries. This can ultimately lead to early management, preventing complications such as joint instability and degenerative changes. The findings reinforce the importance of skillful physical examination and can be incorporated into orthopedic training and clinical decision-making pathways, especially in primary and secondary care centers.
